# Diallyl trisulfide exerts cardioprotection against myocardial ischemia-reperfusion injury in diabetic state, role of AMPK-mediated AKT/GSK-3β/HIF-1α activation

**DOI:** 10.18632/oncotarget.20422

**Published:** 2017-08-24

**Authors:** Liming Yu, Wencheng Di, Xue Dong, Zhi Li, Xiaodong Xue, Jian Zhang, Qi Wang, Xiong Xiao, Jinsong Han, Yang Yang, Huishan Wang

**Affiliations:** ^1^ Department of Cardiovascular Surgery, General Hospital of Shenyang Military Area Command, Shenyang, Liaoning 110016, China; ^2^ Department of Cardiology, Affiliated Drum Tower Hospital of Nanjing University Medical School, Nanjing, Jiangsu 210008, China; ^3^ Department of Pharmacy, General Hospital of Shenyang Military Area Command, Shenyang, Liaoning 110016, China; ^4^ Department of Neurosurgery, General Hospital of Shenyang Military Area Command, Shenyang, Liaoning 110016, China; ^5^ Graduate School, Dalian Medical University, Dalian, Liaoning 116044, China; ^6^ Department of Cardiovascular Surgery, Xijing Hospital, The Fourth Military Medical University, Xi’an, Shaanxi 710032, China; ^7^ Faculty of Life Science, Northwest University, Xi’an, Shaanxi 710069, China; ^8^ Department of Biomedical Engineering, The Fourth Military Medical University, Xi’an, Shaanxi 710032, China

**Keywords:** diallyl trisulfide, diabetes mellitus, myocardial ischemia-reperfusion injury, AMPK, AKT/GSK-3β/HIF-1α signaling

## Abstract

Diallyl trisulfide (DATS), the major active ingredient in garlic, has been reported to confer cardioprotective effects. However, its effect on myocardial ischemia-reperfusion (MI/R) injury in diabetic state and the underlying mechanism are still unknown. We hypothesize that DATS reduces MI/R injury in diabetic state via AMPK-mediated AKT/GSK-3β/HIF-1α activation. Streptozotocin-induced diabetic rats received MI/R surgery with or without DATS (20mg/kg) treatment in the presence or absence of Compound C (Com.C, an AMPK inhibitor, 0.25mg/kg) or LY294002 (a PI3K inhibitor, 5mg/kg). We found that DATS significantly improved heart function and reduced myocardial apoptosis. Additionally, in cultured H9c2 cells, DATS (10μM) also attenuated simulated ischemia-reperfusion injury. We found that AMPK and AKT/GSK-3β/HIF-1α signaling were down-regulated under diabetic condition, while DATS markedly increased the phosphorylation of AMPK, ACC, AKT and GSK-3β as well as HIF-1α expression in MI/R-injured myocardium. However, these protective actions were all blunted by Com.C administration. Additionally, LY294002 abolished the stimulatory effect of DATS on AKT/GSK-3β/HIF-1α signaling without affecting AMPK signaling. While 2-methoxyestradiol (a HIF-1α inhibitor) reduced HIF-1α expression without affecting AKT/GSK-3β signaling. Taken together, these data showed that DATS protected against MI/R injury in diabetic state by attenuating cellular apoptosis via AMPK-mediated AKT/GSK-3β/HIF-1α signaling. Its cardioprotective effect deserves further study.

## INTRODUCTION

Coronary artery disease (CAD) is a major cause of death and disability in developing countries. Although timely reperfusion (such as thrombolytic therapy) is the main treatment to limit infarct size and attenuate cardiac damage, reperfusion itself exacerbates myocardial injury and cellular death, commonly termed as ‘myocardial ischemia-reperfusion (MI/R) injury’ [1, 2]. To make things worse, numerous basic and clinical studies revealed that diabetes mellitus (DM), a common chronic metabolic disease, dramatically increased the risk of CAD, followed by significantly increased cardiac damage and mortality risk [3]. Additionally, previous reports demonstrated that diabetes also compromised the effectiveness of various cardioprotective interventions [4]. During MI/R injury, cardiac damage is more dominant in reperfusion period than that in ischemia period and enhanced cellular apoptosis is deemed to be one of the main causes, especially in diabetic state [3, 4]. Therefore, anti-apoptotic therapy has been shown to reduce MI/R injury [3, 5, 6]. However, there is still a lack of safe and effective therapeutic strategy against diabetic MI/R injury.

Garlic has been used for medicinal purposes for thousands of years. The ancient references to the use of garlic can be traced back to at least the third millennium BCE [7, 8]. During recent years, garlic has attracted more and more attention for its rich health benefits such as anticancer effects, immune-stimulatory effects, lipid lowering effects and, importantly, cardioprotective effects [9-11]. It has been demonstrated that polysulfide compounds, including diallyl sulfide (DAS), diallyl disulfide (DADS) and diallyl trisulfide (DATS), are the major active ingredients in garlic [8]. Among these three ingredients, DATS has been proven to exert most effective cardioprotecitve effect and has been demonstrated to retard cardiac contractile dysfunction in streptozotocin (STZ)-induced diabetic rats [12]. Moreover, Kuo et al. performed a series of experiments which showed that DATS treatment markedly inhibited cardiac apoptosis in hyperglycemic state [13-15]. However, whether DATS regulate cardiomyocyte apoptosis during ischemia-reperfusion injury under diabetic condition and the underlying mechanisms are still unknown.

AMP-activated protein kinase (AMPK) is a crucial defender against ischemic heart disease and cellular stress [16]. AMPK signaling regulates a variety of physiological and pathological activities such as apoptosis, cellular metabolism and energy homeostasis [16]. Importantly, activation of AMPK has been demonstrated to confer a solid protective effect against reperfusion injury [17]. Additionally, AKT signaling serves as its key downstream target [17, 18]. Intriguingly, allicin (the major bioactive compound of crushed garlic), which can be metabolized into diallyl trisulfide, has been found to activate AMPK/Tuberous sclerosis protein 2 (TSC2) signaling pathway in human Hep G2 cells [19]. However, the direct regulatory role of DATS on AMPK signaling in cardiovascular system is poorly defined. Intriguingly, we and others previously found that AKT and its downstream targets Glycogen synthase kinase-3β (GSK-3β) and Hypoxia-inducible factor-1α (HIF-1α) also played key roles in ischemia-reperfusion injury [20-22]. However, whether they contribute to the cardioprotective effect of DATS against diabetic MI/R injury is also unknown.

Therefore, the objectives of this study were to: (1) evaluate whether DATS reduce MI/R injury and preserve cardiac function in diabetic state; (2) determine the roles of AMPK signaling and AKT/GSK-3β/HIF-1α signaling in this process; (3) investigate the detailed mechanisms involved.

## RESULTS

### Cardiac AMPK signaling and AKT/GSK-3β/HIF-1α signaling were down-regulated in type 1 diabetic rat heart

As a key intracellular energy sensor, AMPK plays an essential role in the adaptive response of cardiomyocyte to cellular stress during MI/R injury, especially in diabetic setting [16, 18]. Moreover, AKT/GSK-3β/HIF-1α pathway has been proven to serve as the common pro-survival signal through inhibiting apoptotic signaling cascades [22, 23]. So, in the present study, we firstly evaluated AMPK and AKT/GSK-3β/HIF-1α signaling in the left ventricular of diabetic and non-diabetic rats (Figure 2). Initially, we evaluated the toxic effect of Compound C (Com.C, a selective AMPK inhibitor) and LY294002 (LY, a PI3K inhibitor) on sham-operated diabetic heart (Supplementary Figure 1). Under experimental dosages, Compound C and LY294002 treatment caused no significant changes in the cardiac function, percentage of TUNEL positive nuclei and cellular apoptotic signaling pathway, indicating that exogenous agents caused no significant toxic effects in the present experiment. Then, we found that diabetic myocardium exhibited a significantly reduced phosphorylation levels of AMPK and its downstream effector ACC (Figure 2B and 2C). Additionally, compared with the control group, decreased phosphorylation levels of AKT and GSK-3β as well as HIF-1α expression were also found in the diabetic group (Figure 2D, 2E and 2F). These data were consistent with the previous reports that diabetes mellitus impaired cardiac AMPK signaling and AKT/GSK-3β signaling [24, 25].

**Figure 1:**
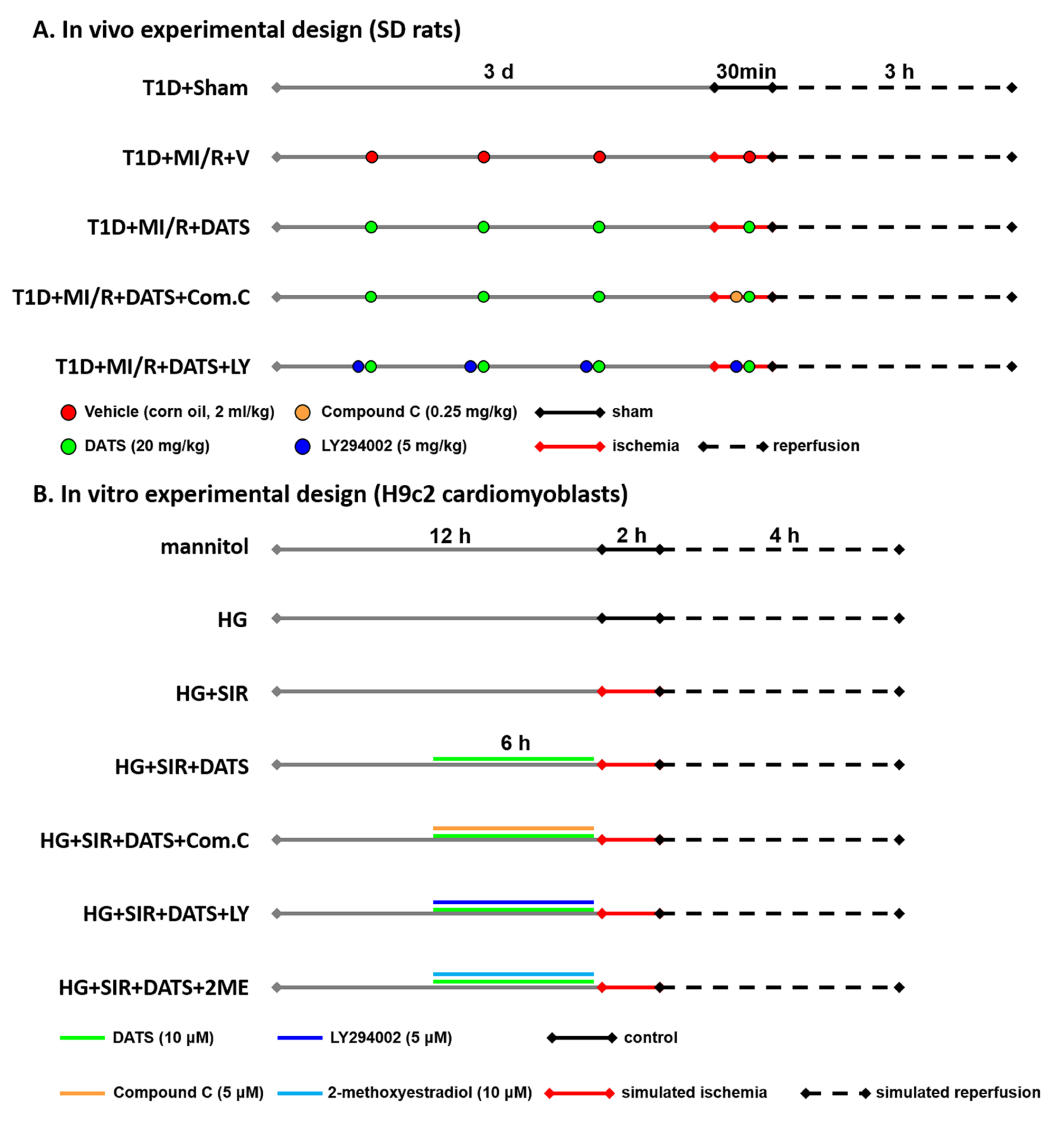
A schematic illustration of *in vivo* and *in vitro* experimental design **(A)**. *in vivo* experimental design; **(B)**. *in vitro* experimental design. T1D, type 1 diabetes; MI/R, myocardial ischemia-reperfusion; DATS, diallyl trisulfide; Com.C, Compound C; LY, LY294002; HG, high glucose; SIR, simulated ischemia-reperfusion; 2ME, 2-methoxyestradiol.

**Figure 2:**
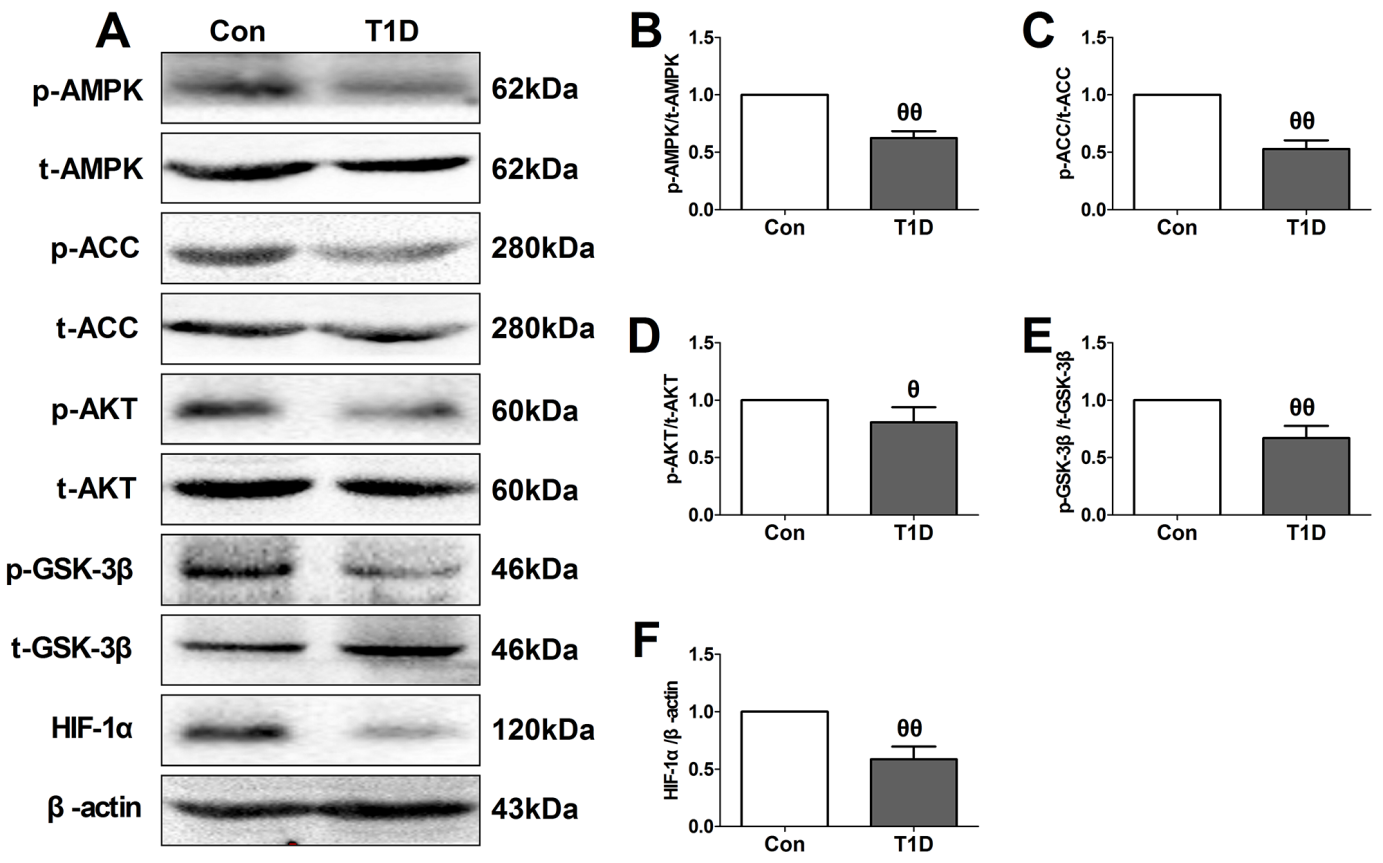
Type 1 diabetic rats showed reduced myocardial AMPK/ACC signaling and AKT/GSK-3β/HIF-1α signaling **(A)**. representative blots; **(B)**. p-AMPK/AMPK ratio; **(C)**. p-ACC/ACC ratio; **(D)**. p-AKT/AKT ratio; **(E)**. p-GSK3β/GSK3β ratio; **(F)**. HIF-1α expression. Data are expressed as mean ± SEM, n = 6 in each group; ^θθ^*P* < 0.01/^θ^*P* < 0.05 vs the Con group. Con, control group; T1D, type 1 diabetic group.

### DATS markedly improved cardiac functional recovery and reduced myocardial infarct size and apoptosis, which was blunted by compound C or LY294002 co-administration

**Figure 3:**
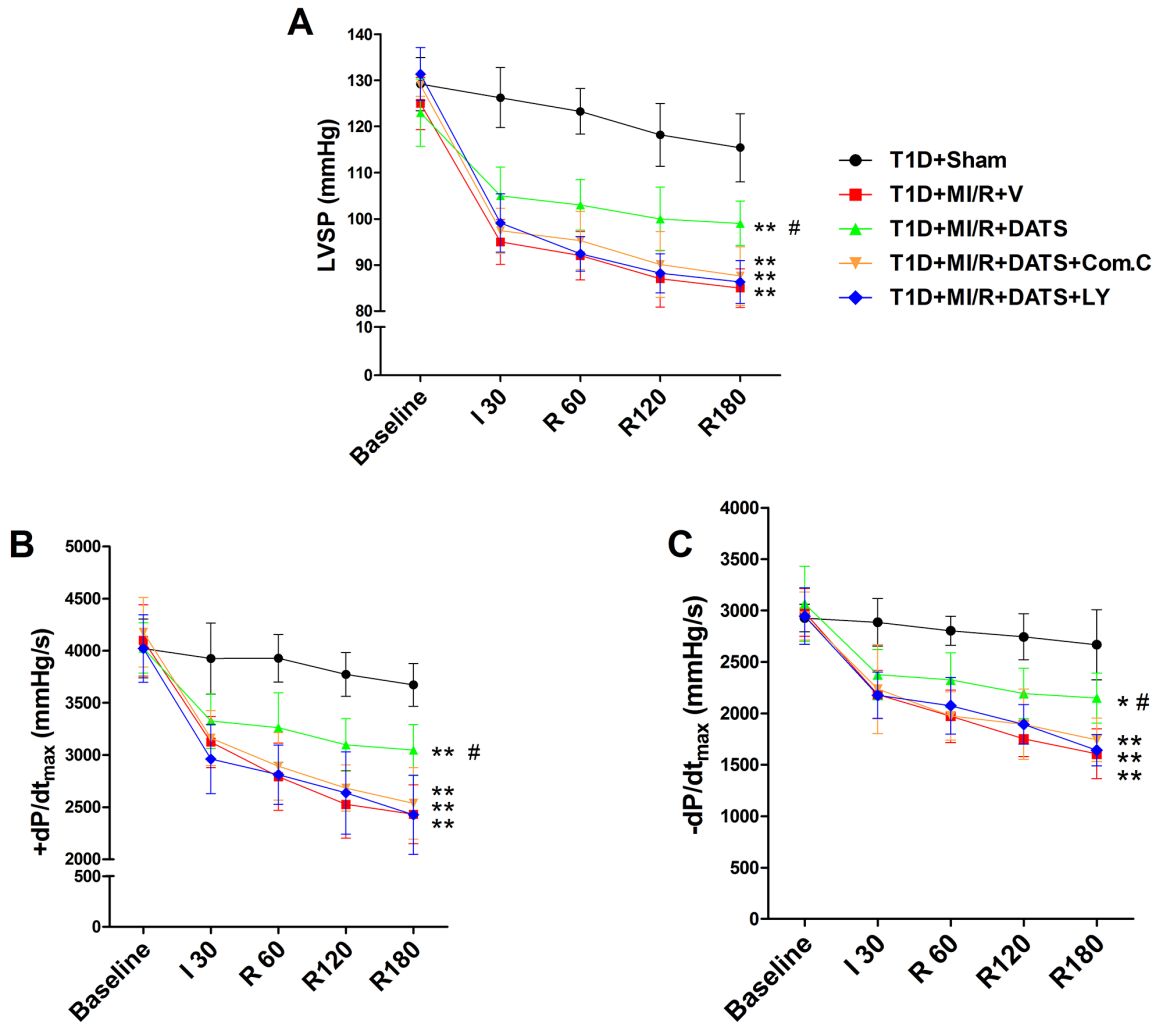
The effect of DATS, Compound C and LY294002 on cardiac function during myocardial ischemia-reperfusion injury **(A)**. Left ventricular systolic pressure (LVSP); **(B)** and **(C)**. The first derivative of left ventricular pressure (+dP/dt_max_ and -dP/dt_max_). Data are expressed as mean ± SEM, n = 6 in each group; ^**^P < 0.01/^*^P < 0.05 vs the T1D+Sham group, ^##^*P* < 0.01/^#^*P* < 0.05 vs the T1D+MI/R+V group. T1D, type 1 diabetes; MI/R, myocardial ischemia-reperfusion; DATS, diallyl trisulfide; Com.C, Compound C; LY, LY294002; I 30, ischemia for 30 min; R 60, 120, 180, reperfusion for 60 min, 120 min, 180 min.

**Figure 4:**
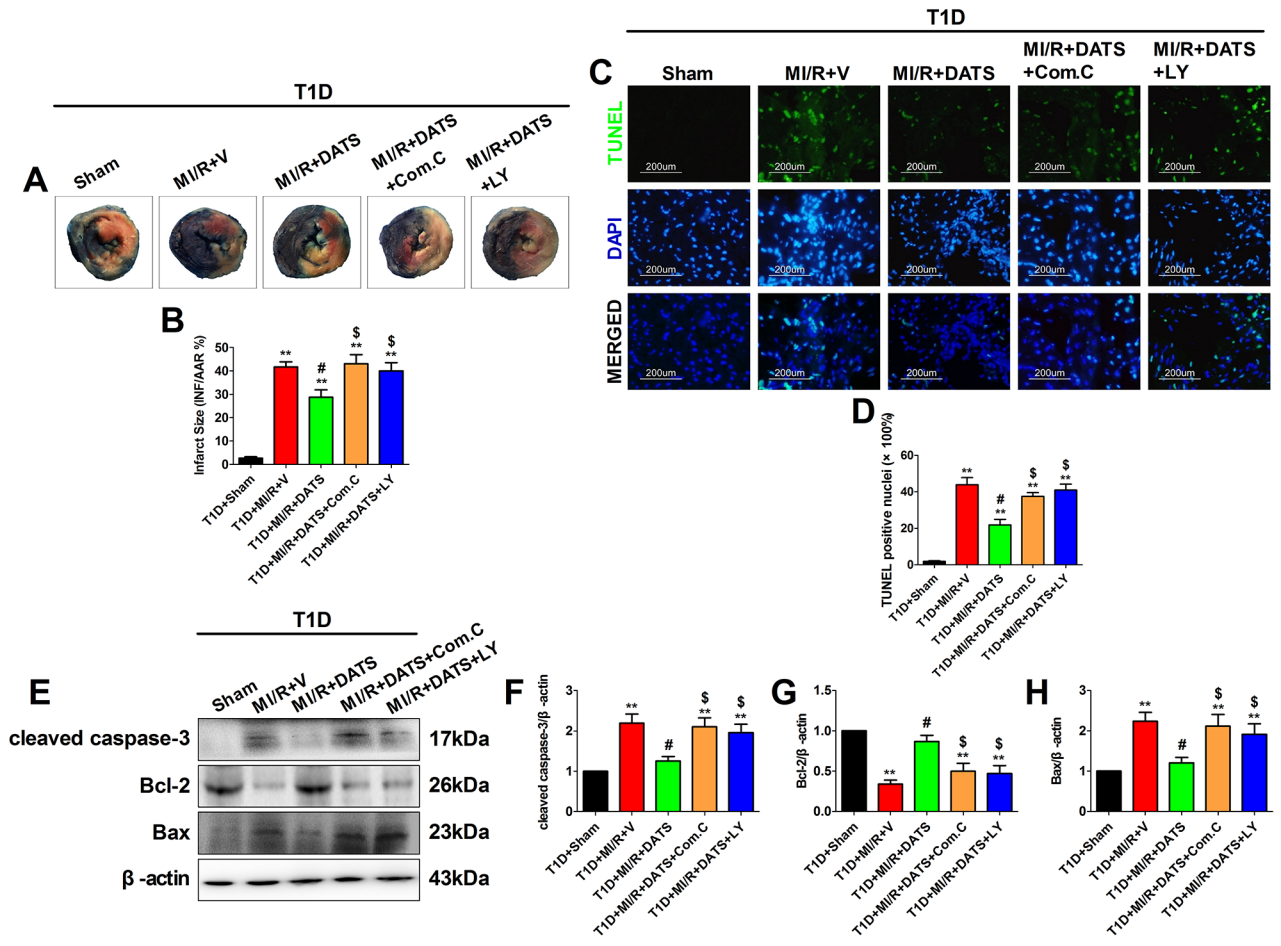
DATS treatment significantly reduced myocardial infarct size and inhibited myocardial apoptosis, which was blunted by Compound C or LY294002 co-administration **(A)** and **(B)**. Myocardial infarct size; **(C)**. Representative photomicrographs of TUNEL staining (×200, bar=200μm). Apoptotic nuclei were stained with TUNEL (Row 1). Total nuclei were stained with DAPI (Row 2). **(D)**. Percentage of TUNEL positive nuclei; **(E)**. representative blots; **(F)**. cleaved caspase-3 expression; **(G)**. Bcl-2 expression; **(H)**. Bax expression. Data are expressed as mean ± SEM, n = 6 in each group; ^**^P < 0.01/^*^P < 0.05 vs the T1D+Sham group, ^##^*P* < 0.01/^#^*P* < 0.05 vs the T1D+MI/R+V group, ^$$^*P* < 0.01/^$^*P* < 0.05 vs the T1D+MI/R+DATS group. T1D, type 1 diabetes; MI/R, myocardial ischemia-reperfusion; DATS, diallyl trisulfide; Com.C, Compound C; LY, LY294002.

DATS, the major active ingredient in garlic, has been demonstrated as a potential cardioprotective compounds [13]. However, whether it confer cardioprotection against MI/R injury in diabetic state and the underlying mechanisms remain poorly defined. As shown in Figure 3, after 3 h of reperfusion, DATS-treated group showed markedly improved left ventricular systolic pressure and first derivative of left ventricular pressure (compared with the T1D+MI/R+V group). However, Compound C or LY294002 co-treatment blunted these effects (compared with the T1D+MI/R+DATS group), indicating that AMPK and AKT signaling might participate in this process. Additionally, we further evaluated the effect of DATS on myocardial infarction and apoptosis. As shown in Figure 4, DATS markedly decreased the infarct size and the percentage of TUNEL positive nuclei. Moreover, DATS-treated group also exhibited markedly increased Bcl-2 expression and decreased cleaved caspase-3 as well as Bax expressions. However, these protective effects were also blunted by Compound C or LY294002 administration, indicating that AMPK and AKT signaling plays an essential role in the anti-apoptotic effect of DATS.

### The effect of DATS, compound C and LY294002 on AMPK and AKT/GSK-3β/HIF-1α signaling in MI/R-injured diabetic rat heart

**Figure 5:**
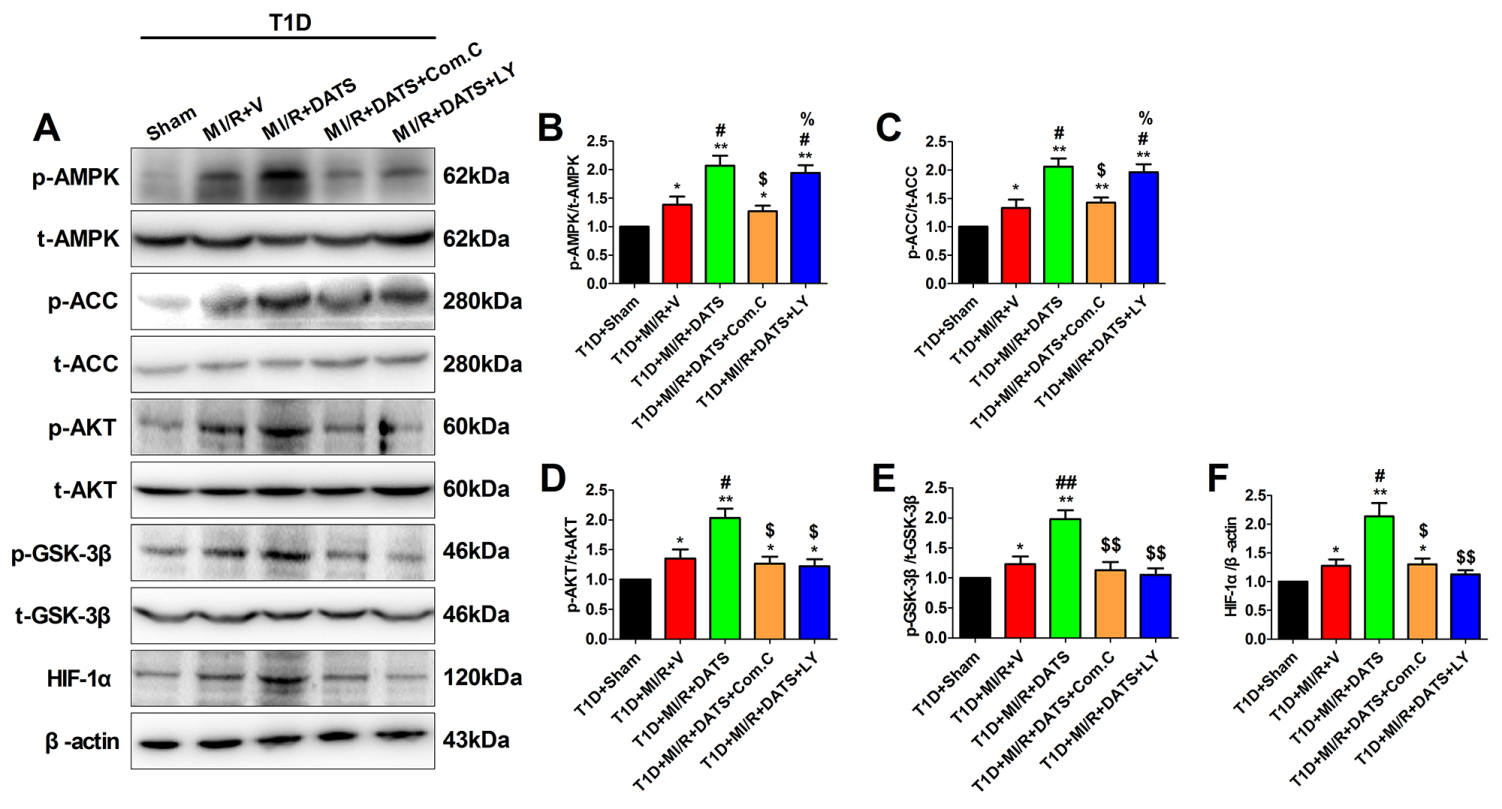
DATS treatment enhanced myocardial AMPK/ACC signaling and AKT/GSK-3β/HIF-1α signaling, which was inhibited by Compound C or LY294002 **(A)**. representative blots; **(B)**. p-AMPK/AMPK ratio; **(C)**. p-ACC/ACC ratio; **(D)**. p-AKT/AKT ratio; **(E)**. p-GSK3β/GSK3β ratio; **(F)**. HIF-1α expression. Data are expressed as mean ± SEM, n = 6 in each group; ^**^P < 0.01/^*^P < 0.05 vs the T1D+Sham group, ^##^*P* < 0.01/^#^*P* < 0.05 vs the T1D+MI/R+V group, ^$$^*P* < 0.01/^$^*P* < 0.05 vs the T1D+MI/R+DATS group. T1D, type 1 diabetes; MI/R, myocardial ischemia-reperfusion; DATS, diallyl trisulfide; Com.C, Compound C; LY, LY294002.

To further elucidate the underlying mechanism, we evaluated AMPK and AKT/GSK-3β/HIF-1α signaling in MI/R-injured diabetic heart. As shown in Figure 5B and 5C, after 3 h of reperfusion, DATS-treated group exhibited significantly increased phosphorylation levels of AMPK and ACC. Meanwhile, DATS also markedly increased the phosphorylation levels of AKT and GSK-3β as well as HIF-1α expression (Figure 5D, 5E and 5F). Moreover, these effects were also abolished by Compound C administration, while LY294002 inhibited AKT/GSK-3β/HIF-1α activation without significantly affecting AMPK signaling. All these data suggested that AMPK and AKT/GSK-3β/HIF-1α signaling played a pivotal role in DATS’s cardioprotecitve actions against diabetic MI/R injury. Additionally, AKT acted as the downstream target of AMPK in this process.

### Compound C attenuated the cytoprotective effect of DATS against SIR injury in high glucose-treated H9c2 cardiomyoblasts

**Figure 6:**
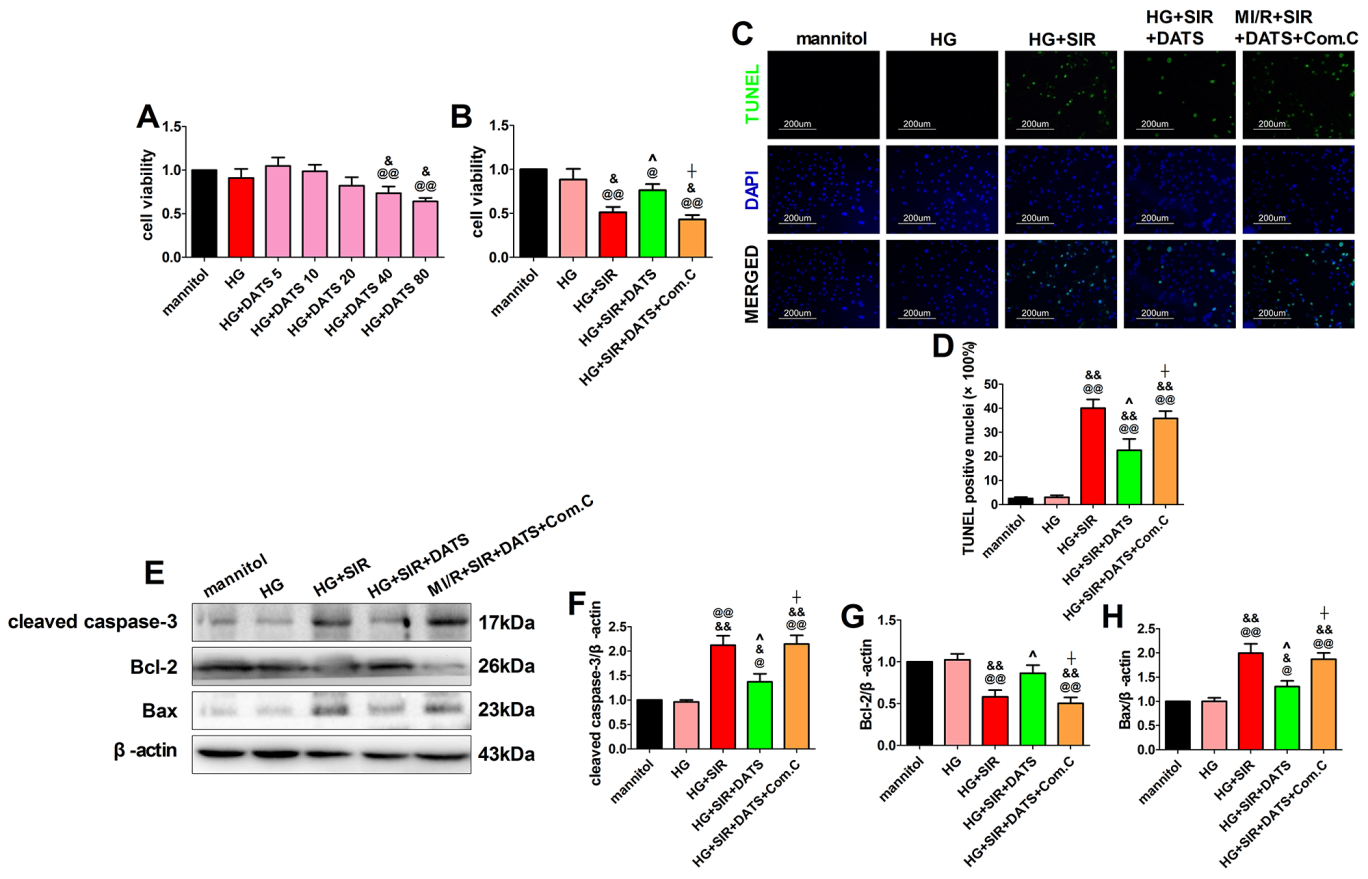
In high glucose-treated H9c2 cardiomyoblasts, DATS effectively attenuated cellular apoptosis, while Compound C bunted this effect **(A)**. High glucose-treated H9c2 cells were incubated in culture medium containing DATS for 6 h at the concentrations of 5, 10, 20, 40 and 80 μmol/L, separately. Then, cell viability was assessed. **(B)**. Cell viability. **(C)**. Representative photomicrographs of TUNEL staining (×200, bar=200μm). Apoptotic nuclei were stained with TUNEL (Row 1). Total nuclei were stained with DAPI (Row 2). **(D)**. Percentage of TUNEL positive nuclei; **(E)**. representative blots; **(F)**. cleaved caspase-3 expression; **(G)**. Bcl-2 expression; **(H)**. Bax expression. Data are expressed as mean ± SEM, n = 6 in each group; ^@@^*P* < 0.01/^@^*P* < 0.05 vs the mannitol group, ^&&^*P* < 0.01/^&^*P* < 0.05 vs the HG group, ^^^^*P* < 0.01/^^^*P* < 0.05 vs the HG+SIR group, ^^^^*P* < 0.01/^^^*P* < 0.05 vs the HG+SIR+DATS group. HG, high glucose; SIR, simulated ischemia-reperfusion; DATS, diallyl trisulfide; Com.C, Compound C.

To further confirm the cardioprotective effect of DATS against MI/R injury in diabetic state and elucidate its mechanisms, we performed *in vitro* experiment using H9c2 cardiomyoblasts. Initially, we evaluated the toxic effect of DATS [5, 10, 20, 40, 80 (in μM)] on high glucose-incubated H9c2 cells. After 12 h of treatment, we found that high glucose treatment slightly decreased the cell viability (compared with the mannitol group), while 40 and 80 μM DATS treatment (for 6 h) significantly reduced cell viability (Figure 6A, compared with the mannitol group). Compared with the HG group, 40 and 80 μM DATS treatment also significantly reduced cell viability, while 20 μM DATS slightly reduced the cell viability (Figure 6A). Based on these experimental data and the previous reports [14], we chose the dose of 10 μM in the present study. We found that DATS conferred a profound protective effect against SIR injury as evidenced by markedly increased cell viability and decreased percentage of TUNEL positive nuclei (Figure 6B, 6C and 6D). Meanwhile, DATS treatment also effectively inhibited the cellular apoptotic signaling by decreasing cleaved caspase-3 and Bax expressions as well as increasing Bcl-2 expression (Figure 6E-6H). Intriguingly, these effects were abolished by Compound C co-incubation (Figure 6), while under the present experimental dosages, Compound C itself did not caused significant changes in cell viability and apoptosis signaling (Supplementary Figure 2), indicating that AMPK signaling contributed greatly to the cytoprotecitve effect of DATS.

### The effect of DATS and Compound C on AMPK signaling and AKT/GSK-3β/HIF-1α signaling in high glucose-treated and SIR-injured H9c2 cells

**Figure 7:**
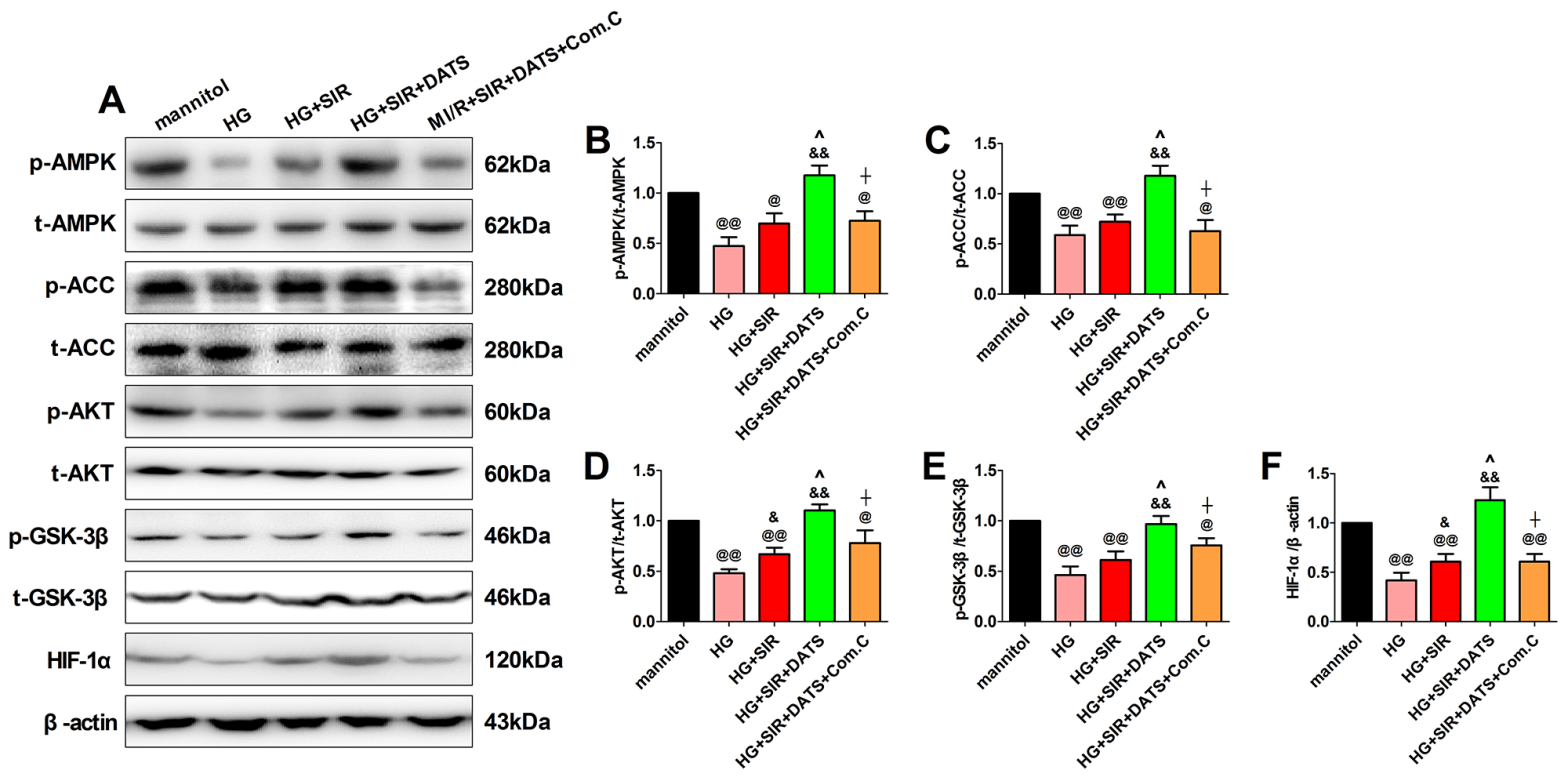
In high glucose-treated H9c2 cardiomyoblasts, DATS markedly enhanced cellular AMPK/ACC signaling and AKT/GSK-3β/HIF-1α signaling, which was blunted by Compound C co-administration **(A)**. representative blots; **(B)**. p-AMPK/AMPK ratio; **(C)**. p-ACC/ACC ratio; **(D)**. p-AKT/AKT ratio; **(E)**. p-GSK3β/GSK3β ratio; **(F)**. HIF-1α expression. Data are expressed as mean ± SEM, n = 6 in each group; ^@@^*P* < 0.01/^@^*P* < 0.05 vs the mannitol group, ^&&^*P* < 0.01/^&^*P* < 0.05 vs the HG group, ^^^^*P* < 0.01/^^^*P* < 0.05 vs the HG+SIR group, ^^^^*P* < 0.01/^^^*P* < 0.05 vs the HG+SIR+DATS group. HG, high glucose; SIR, simulated ischemia-reperfusion; DATS, diallyl trisulfide; Com.C, Compound C.

To further confirm the role of AMPK signaling in DATS’s cytoprotective actions, we measured both the AMPK signaling and AKT/GSK-3β/HIF-1α signaling in the *in vitro* experiment. As shown in Figure 7, we found that 12 h of high glucose incubation markedly inhibited AMPK signaling and AKT/GSK-3β/HIF-1α signaling by reducing the phosphorylation of AMPK, ACC, AKT and GSK-3β as well as the protein expression of HIF-1α (compared with the mannitol group). Next, we found that DATS significantly activated AMPK signaling and AKT/GSK-3β/HIF-1α signaling while Compound C also abolished this effect. All these data provided direct evidence that AMPK signaling contributed greatly to the cytoprotective effect of DATS. Additionally, AKT/GSK-3β/HIF-1α signaling served as the key downstream signaling.

### Effect of LY294002 and 2-methoxyestradiol on SIR injury and cellular AKT/GSK-3β/HIF-1α signaling in high glucose-treated H9c2 cells

**Figure 8:**
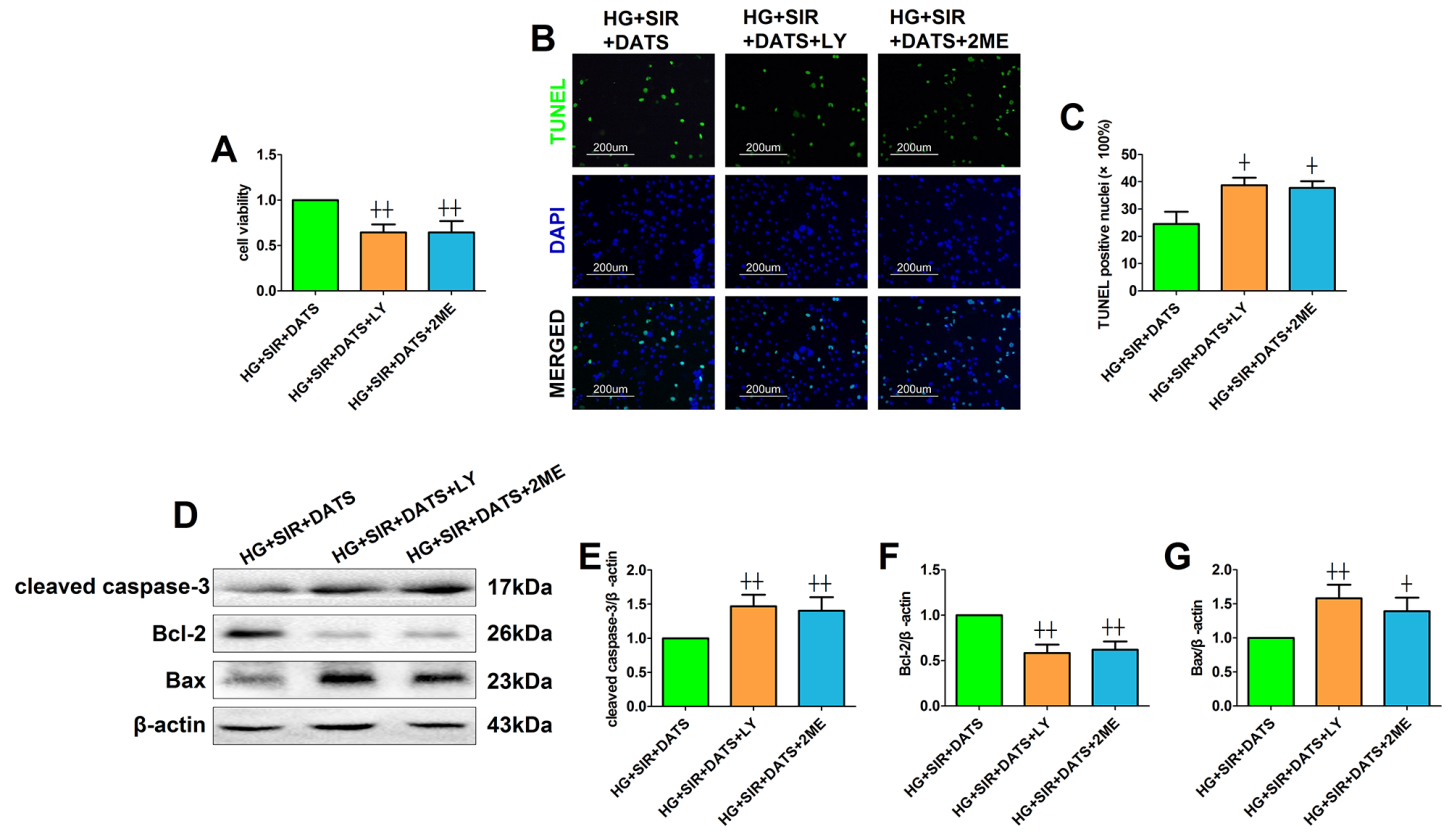
LY294002 and 2-methoxyestradiol inhibited the ameliorative effect of DATS on simulated ischemia reperfusion-induced cellular apoptosis in high glucose-treated H9c2 cells **(A)**. Cell viability. **(B)**. Representative photomicrographs of TUNEL staining (×200, bar=200μm). Apoptotic nuclei were stained with TUNEL (Row 1). Total nuclei were stained with DAPI (Row 2). **(C)**. Percentage of TUNEL positive nuclei; **(D)**. representative blots; **(E)**. cleaved caspase-3 expression; **(F)**. Bcl-2 expression; **(G)**. Bax expression. Data are expressed as mean ± SEM, n = 6 in each group; ^^^^*P* < 0.01/^^^*P* < 0.05 vs the HG+SIR+DATS group. HG, high glucose; SIR, simulated ischemia-reperfusion; DATS, diallyl trisulfide; LY, LY294002; 2ME, 2-methoxyestradiol.

**Figure 9:**
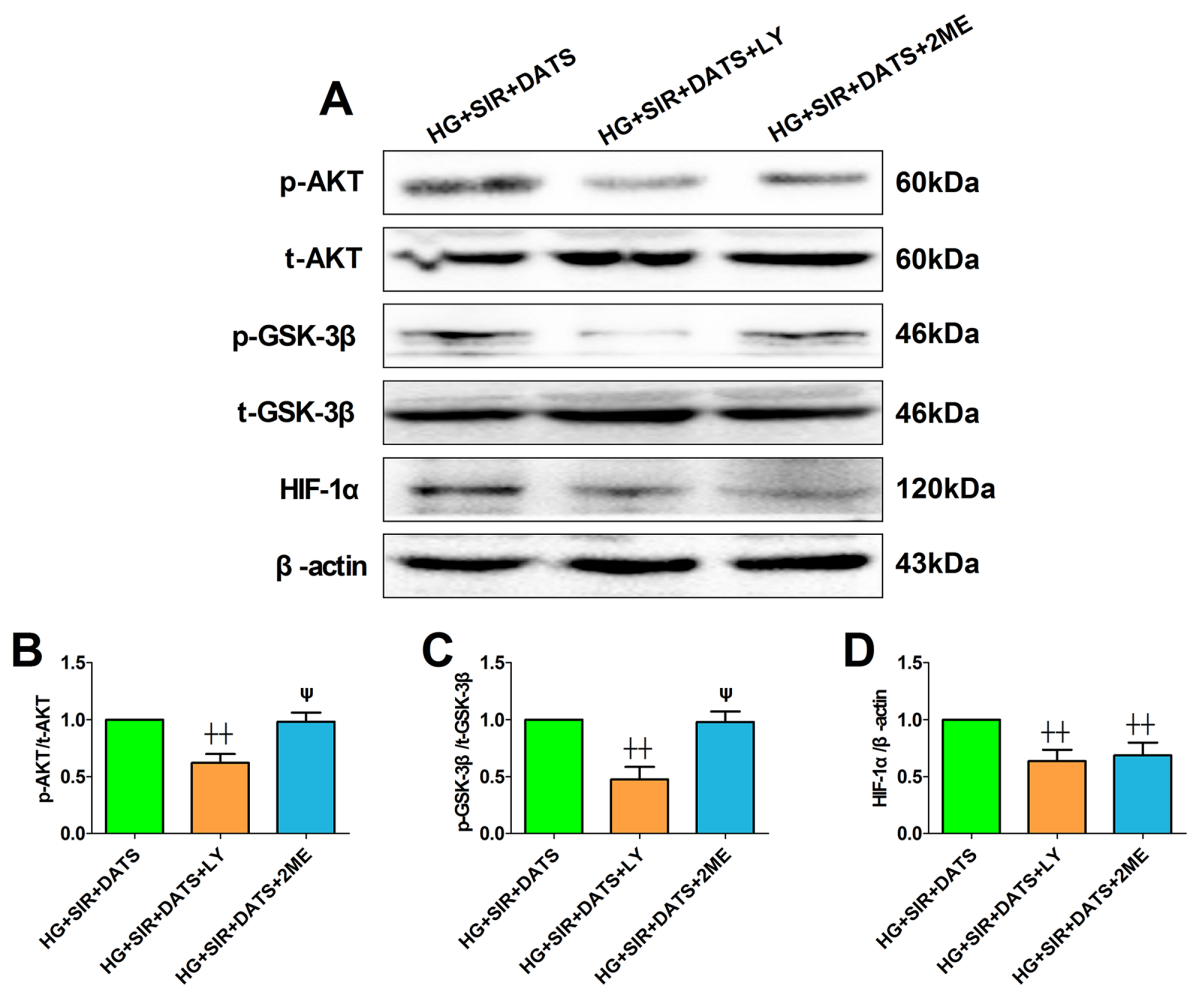
LY294002 blunted cellular AKT/GSK-3β/HIF-1α signaling while 2-methoxyestradiol reduced HIF-1α expression without affecting AKT/GSK-3β signaling **(A)**. representative blots; **(B)**. p-AKT/AKT ratio; **(C)**. p-GSK3β/GSK3β ratio; **(D)**. HIF-1α expression. Data are expressed as mean ± SEM, n = 6 in each group; ^^^^*P* < 0.01/^^^*P* < 0.05 vs the HG+SIR+DATS group, ^ψψ^*P* < 0.01/^ψ^*P* < 0.05 vs the HG+SIR+LY group. HG, high glucose; SIR, simulated ischemia-reperfusion; DATS, diallyl trisulfide; LY, LY294002; 2ME, 2-methoxyestradiol.

Next, we introduced the specific PI3K/AKT inhibitor and HIF-1α inhibitor to further confirm the mechanism. Firstly, we also tested the toxic effect of LY294002 and 2-methoxyestradiol on cell viability and apoptosis in high glucose-treated H9c2 cells (Supplementary Figure 2). Under the present dosages, we did not find a significant difference in cell viability and apoptosis between the inhibitor-treated group and the control group. As shown in Figure 8A, 8B and 8C, LY294002 and 2-methoxyestradiol markedly decreased the cell viability and increased the percentage of TUNEL positive nuclei (compared with the HG+SIR+DATS group). Additionally, the apoptotic signaling was also re-activated in the inhibitor-treated groups (Figure 8D-8G). As shown in Figure 9, LY294002 blunted cellular AKT/GSK-3β/HIF-1α signaling as evidenced by reduced phosphorylation levels of AKT and GSK-3β as well as deceased HIF-1α expression (compared with the HG+SIR+DATS group). However, 2-methoxyestradiol reduced HIF-1α expression without affecting AKT/GSK-3β signaling, indicating that HIF-1α served as the downstream target of AKT/GSK-3β signaling.

## DISCUSSION

In the present study, we evaluated the cardioprotective effect of DATS on MI/R injury in type 1 diabetic state. Our *in vivo* and *in vitro* data showed that DATS treatment significantly improved cardiac functional recovery and inhibited cellular apoptosis after myocardial reperfusion. In addition, the crucial roles of AMPK and its downstream target AKT/GSK-3β/HIF-1α signaling were revealed. To the best of our knowledge, this is the first study disclosing the potential effect of DATS on diabetic MI/R injury and its underlying mechanisms.

During recent years, DATS has attracted more and more attention as a therapeutic agent against plenty of diseases, including cancer, infections, metabolic syndrome and cardiovascular disease [26-28]. Several studies have investigated its myocardial protective actions. Predmore et al. firstly reported that DATS significantly reduced MI/R injury in non-diabetic mice by limiting infarct size and improving cardiac contractile function [29]. They further demonstrated that DATS could act as a H_2_S donor and preserved endogenous H_2_S levels, thus activating endothelial nitric oxide synthase (eNOS) and increasing the bioavailability of nitric oxide (NO) [29]. In addition, a series of study by Kuo et al. showed that DATS inhibited high glucose-induced cardiomyocyte apoptosis by reducing JNK/NF-κB axis and activating PI3K/AKT signaling [13, 14]. Intriguingly, one of their recent study also demonstrated that AKT signaling played a pivotal role in the cytoprotective actions of DATS in hyperglycemic setting [15]. However, whether DATS reduces MI/R injury in diabetic or hyperglycemic state and the role of AKT signaling in this process are still unknown. In the present study, our *in vivo* and *in vitro* results demonstrated that DATS effectively attenuated MI/R injury in type 1 diabetic state as evidenced by improved heart function and reduced cellular apoptosis. Moreover, we determined the potential role of AKT signaling and further investigated its upstream and downstream target.

AMPK has been confirmed as the crucial modulator of cellular metabolism and stress in the cardiomyocyte [18]. Activation of AMPK regulates the transport and metabolism of fatty acids and glucose to ameliorate cardiac damage [16, 24]. Plenty of pathophysiological conditions including heat shock, starvation, oxidative damage and ischemia-reperfusion injury can activate AMPK signaling, thus enhancing glycolysis and fatty acid oxidation [16, 18]. However, in diabetic condition, AMPK signaling is found to be down-regulated in adipose tissue, liver and even in myocardium [16, 30, 31]. One study showed that high-fat diet-treated C57BL/6 mice (6 weeks) exhibited a significantly reduced myocardial AMPK activity and enhanced myocardial inflammatory response [31]. Another study by Xie et al. also found a markedly reduced AMPK activation in STZ-induced type 1 diabetic mice [32]. Consistently, our *in vivo* and *in vitro* data showed that diabetes or high glucose incubation markedly decreased the phosphorylation levels of AMPK and its downstream effector ACC. Importantly, previous literature demonstrated that reduced AMPK signaling was the main cause of cardiovascular disease and constituted a crucial mechanism for aggravated myocardial ischemic injury in diabetic condition [33]. Therefore, AMPK signaling in the ischemic heart has been deemed as an important therapeutic target to ameliorate MI/R injury in diabetic condition [34-36]. One of the previous reports showed that allicin, which can be metabolized into diallyl trisulfide, could activate AMPK signaling in Hep G2 human liver cancer cells [19]. However, in cardiovascular system, the regulatory role of DATS on AMPK has not been disclosed. In this study, we firstly found that DATS significantly enhanced AMPK signaling in MI/R-injured heart. Meanwhile, co-treatment with Compound C, the selective inhibitor of AMPK, not only inhibited AMPK activation, but also blocked the cardioprotective effect of DATS. These data indicate that AMPK signaling is the novel downstream target of DATS. Moreover, we found that DATS also enhanced cardiac AKT/GSK-3β/HIF-1α signaling while this effect was abolished by Compound C administration, suggesting that AKT/GSK-3β/HIF-1α signaling served as the downstream target of AMPK signaling.

GSK-3β, a ubiquitously expressed serine/threonine kinase, is deemed as a pivotal regulator of mitochondrial function as well as cellular apoptosis during MI/R injury [37, 38]. Previous studies indicated that GSK-3β could be modulated by phosphorylation of its specific amino acid residues, serine 9 and tyrosine 216 [38]. Additionally, AKT has been confirmed as the key upstream signaling that phosphorylates GSK-3β at serine 9, leading to the inactivation of GSK-3β, which further inhibits cardiomyocyte apoptosis and confers cardioprotective effects [39]. Previously, we and others found that Akt/GSK-3β cascade acted as an intracellular compensatory feedback regulator that inhibited apoptosis and preserved cardiac function in response to MI/R injury [21, 40]. Modulation of Akt/GSK-3β signaling has also been confirmed as the key mechanism of other cardioprotective agents [21, 35, 41, 42]. In the present study, our *in vivo* and *in vitro* data showed that diabetes significantly down-regulated AKT/GSK-3β signaling in sham-operated heart while DATS markedly enhanced the phosphorylation levels of AKT and GSK-3β. However, Compound C administration markedly inhibited the modulatory effect of AKT/GSK-3β signaling by DATS, while LY294002 significantly blunted AKT/GSK-3β signaling without affecting the AMPK signaling, indicating that AKT/GSK-3β signaling acted as the downstream target of AMPK in the current circumstance.

Previously, AKT/GSK-3β activation has also been linked to increased HIF-1α expression in many studies [43-45]. HIF-1α is a key modulator of hypoxia which enhances the transcription of multiple hypoxia-sensitive genes, including vascular endothelial growth factor (VEGF) and erythropoietin (EPO). Plenty of studies have demonstrated that HIF-1α participated in various cell activity such as cell proliferation, angiogenesis and glucose metabolism [46, 47]. During MI/R injury, HIF-1α is also demonstrated to play a key role in cardioprotection against reperfusion injury [47, 48]. Liu et al. demonstrated that dihydromyricetin, one of the most abundant components in vine tea, markedly inhibited cellular apoptosis-induced by MI/R injury via PI3K/AKT and HIF-1α signaling [49]. Another study by Du et al. found that renalase, an enzyme that can metabolize catecholamine, served as a novel downstream effector of HIF-1α and protected against MI/R injury [50]. However, previous literature also implicated that diabetes mellitus could significantly impair the myocardial HIF signaling, which further interfered with the intrinsic pro-survival signaling pathways and increased myocardial infarct size [51-53]. Consistently, we found a markedly reduced HIF-1α protein expression in diabetic myocardium or high glucose-treated H9c2 cells. Importantly, we also demonstrated that HIF-1α signaling was activated by DATS administration, while inhibition of HIF-1α abolished the cardiprotective effect of DATS without affecting the phosphorylation levels of AMPK, AKT and GSK-3β. These data all suggest that AMPK-mediated AKT/GSK-3β/HIF-1α activation is the key mechanism in DATS’s cardioprotecitve effect in diabetic state. To the best of our knowledge, this is also the first study linking DATS with HIF-1α in cardiovascular system. Importantly, our study also investigated the upstream and downstream relationship between AMPK signaling and AKT/GSK-3β/HIF-1α signaling in this process.

This study proposed novel therapeutic strategy against MI/R injury under diabetic condition. However, there are still more to investigate behind these data. Firstly, under physiological condition, the heart predominantly uses fatty acids as fuel since fatty acids produce more ATP per molecule of substrate than glucose [16]. However, when oxygen supply limits (during myocardial ischemic event), a shift in the myocardial metabolism from fatty acid to glucose is initiated as it is more oxygen efficient and prevents deleterious effects. Previously, activation of AMPK/AKT signaling has also been demonstrated to enhance myocardial glucose uptake thus alleviating MI/R injury [54, 55]. So, the effect of diallyl trisulfide on glucose utilization during MI/R injury deserves further study. Secondly, as type 2 diabetes is the most common form of diabetes, accounting for more than 90% of all cases, it is of great importance to further investigate the potential role of DATS and AMPK-mediated AKT/GSK-3β/HIF-1α signaling pathway under type 2 diabetic condition. Thirdly, several publications have revealed that the anti-oxidative property of DATS contributed greatly to its cardiovascular beneficial effects [14, 15]. For example, Tsai et al. found that DATS attenuated hyperglycemia-induced ROS-mediated apoptosis by activating PI3K/Akt/Nrf2 signaling, which further enhanced Nrf2-mediated antioxidant enzymes in cardiomyocytes exposed to hyperglycemia [14]. Further basic and clinical study is required to evaluate whether DATS confer a similar effect against MI/R injury in diabetic condition as well as the underlying mechanisms.

Taken together, the current experimental data showed that type 1 diabetes impaired myocardial AMPK signaling and AKT/GSK-3β/HIF-1α signaling. DATS treatment reduced MI/R injury in diabetic state by attenuating cellular apoptosis via reactivation of AMPK-mediated AKT/GSK-3β/HIF-1α signaling pathway. This study not only provided novel insights into the etiogenesis of cardiovascular complications under diabetic condition, but also highlighted DATS’s cardioprotective effect for the diabetic patients with ischemic heart disease.

## MATERIALS AND METHODS

### Ethics statement

All experimental procedures were approved by the Animal Care Committee of General Hospital of Shenyang Military Area Region and conformed to Guide for the Care and Use of Laboratory Animals (US National Institutes of Health, Publication No. 85-23, revised 1996). All necessary efforts were made to minimize suffering.

### Animals

Healthy male Sprague-Dawley (SD) rats (Eight-week-old, 180-210 g) were purchased from the Experimental Animal Center of General Hospital of Shenyang Military Area Region. The rats involved in this experiment were fed with standard diet and water under standard animal room conditions. Before the experiment, the animals were housed for one week to familiarize with the surroundings.

### Reagents

Diallyl trisulfide was purchased from LKT Laboratories (St. Paul, MN, USA). Streptozotocin (STZ), 4’, 6-diamino-2-phenylindole (DAPI) and Compound C (Com.C) were obtained from Sigma-Aldrich (St. Louis, MO, USA). Sodium pentobarbital, Evans blue (EB) and triphenyltetrazolium chloride (TTC) were purchased from Solarbio Life Sciences (Beijing, China). 2-methoxyestradiol (2ME) was purchased from Selleck Chemicals (Houston, TX, USA). LY294002 and BCA Protein Assay Kit were purchased from Beyotime Biotechnology (Shanghai, China). Terminal deoxynucleotidyltransferase-mediated dUTP nick end labeling (TUNEL) assay kit was purchased from Roche (Mannheim, Germany). H9c2 cardiomyoblasts were obtained from Tiancheng biotechnology (Shanghai, China). The primary antibodies against p-AMPK (Thr172), AMPK, cleaved caspase-3, Bcl-2 and Bax were obtained from Cell Signaling Technology (Boston, MA, USA). The primary antibodies against p-ACC (Ser79), ACC, p-AKT (Thr308), AKT, p-GSK3β (Ser9), GSK3β, HIF-1α and β-actin were purchased from Santa Cruz Biotechnology (Dallas, TX, USA). The secondary antibodies were purchased from the Zhongshan Company (Beijing, China).

### Diabetes induction

Type 1 diabetic rat model was induced as described in our recent study [25]. In brief, low-dose STZ (40 mg/kg/d, i.p., dissolved in 0.1 mol/l citrate buffer) was administered intraperitoneally for 3 consecutive days. Before the injection, the rats were fasted overnight. Diabetes was confirmed by fasting plasma glucose readings of ≥ 11.1 mmol/L. We also performed oral or intraperitoneal glucose tolerance test to further confirm the diabetic model.

### *In vivo* experimental design

Male SD rats were randomly assigned to 7 experimental groups (Figure 1), (1) non-diabetic control group (Con); (2) type 1 diabetic group (T1D); (3) T1D+Sham; (4) T1D+MI/R+vehicle (V); (5) T1D+MI/R+DATS; (6) T1D+MI/R+DATS+Compound C (Com.C); (7) T1D+MI/R+DATS+LY294002 (LY). Diallyl trisulfide (20 mg/kg, dissolved in corn oil) was orally taken for 3 days before the myocardial ischemia-reperfusion surgery. After 20 min of ischemia, it was administered once again. Corn oil was used as the vehicle at a dose of 2 ml/kg/d. Compound C (0.25 mg/kg) was intravenously treated after 10 min of ischemia. LY294002 was intraperitoneally administered for 3 days before the surgery and once again after 10 min of ischemia. The dose of diallyl trisulfide was chosen based on the previous reports [15]. The dose of Compound C and LY294002 was chosen based on our recent studies [21, 25]. Additionally, the potential toxic effect of the exogenous reagents on the heart was also evaluated (Supplementary Figure 1).

### Myocardial ischemia-reperfusion protocol and cardiac function assessment

Myocardial ischemia-reperfusion operation was performed as described previously [56]. The rats were anesthetized by sodium pentobarbital (40 mg/kg, i.p.) and ventilated via a tracheal intubation on a rodent ventilator (Taimeng technology, Chengdu, China). Following a left lateral thoracotomy, the heart was exposed and a 6-0 silk suture was placed around the left coronary artery. Myocardial ischemia was initiated by ligating the suture, while the rats in the sham group received threading but not ligation. After 30 min of coronary occlusion, the ligature was released and the rats received reperfusion for 3 h. During the operation, left ventricular function of the rats was continuously recorded using a hemodynamic analyzing system (Taimeng technology, Chengdu, China) [25]. First derivative of left ventricular pressure (+dP/dt_max_ and -dP/dt_max_) and Left ventricular systolic pressure (LVSP) were continuously recorded at baseline, ischemia for 30 min and reperfusion for 1, 2, and 3 h.

### Cell culture and *in vitro* experimental design

High glucose treatment was carried out as described by previous studies [57]. H9c2 cardiomyoblasts were cultured in Dulbecco’s modified essential medium (DMEM) supplemented with 10% fetal bovine serum (FBS), 100 U/mL penicillin and 100 mg/mL streptomycin in humidified atmosphere (5% CO_2_) at 37 °C. *in vitro* experiment was illustrated in Figure 1. Initially, the cardiomyoblasts were cultured in normal glucose (5.5 mM) medium. Then, the cells were incubated in normal medium (5.5 mM D-glucose plus 27.5 mM mannitol) or high glucose medium (33 mM D-glucose) for 12 h with or without DATS administration (10 μM, 6 h). Compound C (5 μM), LY294002 (5 μM) or 2-methoxyestradiol (10 μM) was applied in the medium with the administration of DATS in different group. After these treatment, the cells were subjected to simulated ischemia-reperfusion injury. The dosages of DATS and the inhibitors are chosen based on previous studies [21, 22, 58, 59]. Also, we assessed the potential toxic effect of the exogenous reagents on the cells (Supplementary Figure 2).

### Simulated ischemia-reperfusion protocol and cell viability measurement

Simulated ischemia-reperfusion was carried out strictly according to our previous reports [5]. In brief, H9c2 cardiomyoblasts were incubated in the ischemic buffer containing 137 NaCl, 12 KCl, 0.9 CaCl_2_ and 0.49 MgCl_2_ (mM). Additionally, 10 deoxyglucose, 20 lactate, 0.75 sodium dithionate and 4 HEPES (mM) were also supplemented in this buffer. The buffer pH was finally adjusted to 6.5 and the cells were subjected to simulated ischemia for 2 h in the standard humidified atmosphere (5% CO2) at 37 °C. Then, the cells were returned to normal culture medium for 4 h of simulated reperfusion. Before the simulated ischemia treatment, the cells received different treatment.

Cell viability was measured as previously described [60]. The cells were initially seeded in 96-well culture plates (1 × 10^4^ per well). After different treatment, 10 μL CCK-8 solution (1/10 dilution) was added to each well and incubated for 2 h. A microtiter plate reader (SpectraMax 190, Molecular Device, USA) was employed to determine the absorbance. The cell viability was expressed by dividing the optical density of testing samples with that of the mannitol group.

### Myocardial apoptosis and infarction measurement

TUNEL staining was performed strictly following the manufacturer’s instructions as described by our previous reports [25]. The percentage of TUNEL positive nuclei was presented as the number of positively stained apoptotic nuclei (green staining)/the total number of nuclei (blue staining) counted × 100%. Myocardial infarct size was evaluated by Evans Blue-TTC double-staining technique and analyzed using a digital imaging system as described before [61]. The myocardial infarct size was presented as a percentage of infarct area (INF) over total area at risk (AAR) (INF/AAR × 100%).

### Western blotting

Total protein in cardiac tissue and H9c2 cardiomyoblasts was harvested using protein lysis buffer and determined by BCA Protein Assay Kit. Equal amounts of protein (30 μg) was subjected to the 10 % SDS-PAGE gel and then transferred to the polyvinylidene difluoride (PVDF) membranes following with normal western blotting procedures [61]. Protein bands were detected and analyzed using Bio-Image Analysis System (Bio-Rad, Richmond, CA, USA).

### Statistical analysis

Results are presented as means ± SEM. Differences were compared by Student’s t test or ANOVA followed by Bonferroni correction for post hoc t test, where appropriate (GraphPad Prism software, San Diego, CA, USA). Statistically significant difference was considered at a value of *P* < 0.05.

## SUPPLEMENTARY MATERIALS FIGURES



## Abbreviations

2ME: 2-methoxyestradiol
AAR: area at risk
AMPK: AMP-activated protein kinase
CAD: coronary artery disease
Com.C: Compound C
DAPI: 4’, 6-diamino-2-phenylindole
DAS: diallyl sulfide
DADS: diallyl disulfide
DATS: diallyl trisulfide
DM: diabetes mellitus
DMEM: Dulbecco’s modified essential medium
EB: Evans blue
eNOS: endothelial nitric oxide synthase
EPO: erythropoietin
FBS: fetal bovine serum
GSK-3β: Glycogen synthase kinase-3β
HIF-1α: Hypoxia-inducible factor-1α
INF: infarct area
LY: LY294002
MI/R: myocardial ischemia-reperfusion
NO: increasing the bioavailability of nitric oxide
PVDF: polyvinylidene difluoride
STZ: streptozotocin
TSC2: Tuberous sclerosis protein 2
TTC: triphenyltetrazolium chloride
TUNEL: terminal deoxynucleotidyltransferase-mediated dUTP nick end labeling
VEGF: vascular endothelial growth factor

## References

[1] Gewirtz H, Dilsizian V (2017). Myocardial Viability: Survival Mechanisms and Molecular Imaging Targets in Acute and Chronic Ischemia. Circ Res.

[2] Parviz Y, Vijayan S, Lavi S (2017). A review of strategies for infarct size reduction during acute myocardial infarction. Cardiovasc Revasc Med.

[3] Lejay A, Fang F, John R, Van JA, Barr M, Thaveau F, Chakfe N, Geny B, Scholey JW (2016). Ischemia reperfusion injury, ischemic conditioning and diabetes mellitus. J Mol Cell Cardiol.

[4] Schnell O, Cappuccio F, Genovese S, Standl E, Valensi P, Ceriello A (2013). Type 1 diabetes and cardiovascular disease. Cardiovasc Diabetol.

[5] Yu L, Liang H, Dong X, Zhao G, Jin Z, Zhai M, Yang Y, Chen W, Liu J, Yi W, Yang J, Yi D, Duan W, Yu S (2015). Reduced silent information regulator 1 signaling exacerbates myocardial ischemia-reperfusion injury in type 2 diabetic rats and the protective effect of melatonin. J Pineal Res.

[6] Shi Z, Fu F, Yu L, Xing W, Su F, Liang X, Tie R, Ji L, Zhu M, Yu J, Zhang H (2015). Vasonatrin peptide attenuates myocardial ischemia-reperfusion injury in diabetic rats and underlying mechanisms. Am J Physiol Heart Circ Physiol.

[7] Bradley JM, Organ CL, Lefer DJ (2016). Garlic-Derived Organic Polysulfides and Myocardial Protection. J Nutr.

[8] Khatua TN, Adela R, Banerjee SK (2013). Garlic and cardioprotection: insights into the molecular mechanisms. Can J Physiol Pharmacol.

[9] Padiya R, Banerjee SK (2013). Garlic as an anti-diabetic agent: recent progress and patent reviews. Recent Pat Food Nutr Agric.

[10] Yeh YY, Liu L (2001). Cholesterol-lowering effect of garlic extracts and organosulfur compounds: human and animal studies. J Nutr.

[11] Yi L, Su Q (2013). Molecular mechanisms for the anti-cancer effects of diallyl disulfide. Food Chem Toxicol.

[12] Ou HC, Tzang BS, Chang MH, Liu CT, Liu HW, Lii CK, Bau DT, Chao PM, Kuo WW (2010). Cardiac contractile dysfunction and apoptosis in streptozotocin-induced diabetic rats are ameliorated by garlic oil supplementation. J Agric Food Chem.

[13] Kuo WW, Wang WJ, Tsai CY, Way CL, Hsu HH, Chen LM (2013). Diallyl trisufide (DATS) suppresses high glucose-induced cardiomyocyte apoptosis by inhibiting JNK/NFkappaB signaling via attenuating ROS generation. Int J Cardiol.

[14] Tsai CY, Wang CC, Lai TY, Tsu HN, Wang CH, Liang HY, Kuo WW (2013). Antioxidant effects of diallyl trisulfide on high glucose-induced apoptosis are mediated by the PI3K/Akt-dependent activation of Nrf2 in cardiomyocytes. Int J Cardiol.

[15] Tsai CY, Wen SY, Shibu MA, Yang YC, Peng H, Wang B, Wei YM, Chang HY, Lee CY, Huang CY, Kuo WW (2015). Diallyl trisulfide protects against high glucose-induced cardiac apoptosis by stimulating the production of cystathionine gamma-lyase-derived hydrogen sulfide. Int J Cardiol.

[16] Qi D, Young LH (2015). AMPK: energy sensor and survival mechanism in the ischemic heart. Trends Endocrinol Metab.

[17] Kubli DA, Gustafsson AB (2014). Cardiomyocyte health: adapting to metabolic changes through autophagy. Trends Endocrinol Metab.

[18] Daskalopoulos EP, Dufeys C, Bertrand L, Beauloye C, Horman S (2016). AMPK in cardiac fibrosis and repair: Actions beyond metabolic regulation. J Mol Cell Cardiol.

[19] Chu YL, Ho CT, Chung JG, Rajasekaran R, Sheen LY (2012). Allicin induces p53-mediated autophagy in Hep G2 human liver cancer cells. J Agric Food Chem.

[20] Kalakech H, Tamareille S, Pons S, Godin-Ribuot D, Carmeliet P, Furber A, Martin V, Berdeaux A, Ghaleh B, Prunier F (2013). Role of hypoxia inducible factor-1alpha in remote limb ischemic preconditioning. J Mol Cell Cardiol.

[21] Yu L, Li B, Zhang M, Jin Z, Duan W, Zhao G, Yang Y, Liu Z, Chen W, Wang S, Yang J, Yi D, Liu J, Yu S (2016). Melatonin reduces PERK-eIF2alpha-ATF4-mediated endoplasmic reticulum stress during myocardial ischemia-reperfusion injury: role of RISK and SAFE pathways interaction. Apoptosis.

[22] Huang X, Zuo L, Lv Y, Chen C, Yang Y, Xin H, Li Y, Qian Y (2016). Asiatic Acid Attenuates Myocardial Ischemia/Reperfusion Injury via Akt/GSK-3beta/HIF-1alpha Signaling in Rat H9c2 Cardiomyocytes. Molecules.

[23] Chaanine AH, Hajjar RJ (2011). AKT signalling in the failing heart. Eur J Heart Fail.

[24] Horman S, Beauloye C, Vanoverschelde JL, Bertrand L (2012). AMP-activated protein kinase in the control of cardiac metabolism and remodeling. Curr Heart Fail Rep.

[25] Yu L, Gong B, Duan W, Fan C, Zhang J, Li Z, Xue X, Xu Y, Meng D, Li B, Zhang M, Bin Z, Jin Z, Yu S, Yang Y, Wang H (2017). Melatonin ameliorates myocardial ischemia/reperfusion injury in type 1 diabetic rats by preserving mitochondrial function: role of AMPK-PGC-1alpha-SIRT3 signaling. Sci Rep.

[26] Antony ML, Singh SV (2011). Molecular mechanisms and targets of cancer chemoprevention by garlic-derived bioactive compound diallyl trisulfide. Indian J Exp Biol.

[27] Hosseini A, Hosseinzadeh H (2015). A review on the effects of Allium sativum (Garlic) in metabolic syndrome. J Endocrinol Invest.

[28] Bayan L, Koulivand PH, Gorji A (2014). Garlic: a review of potential therapeutic effects. Avicenna J Phytomed.

[29] Predmore BL, Kondo K, Bhushan S, Zlatopolsky MA, King AL, Aragon JP, Grinsfelder DB, Condit ME, Lefer DJ (2012). The polysulfide diallyl trisulfide protects the ischemic myocardium by preservation of endogenous hydrogen sulfide and increasing nitric oxide bioavailability. Am J Physiol Heart Circ Physiol.

[30] Sriwijitkamol A, Coletta DK, Wajcberg E, Balbontin GB, Reyna SM, Barrientes J, Eagan PA, Jenkinson CP, Cersosimo E, DeFronzo RA, Sakamoto K, Musi N (2007). Effect of acute exercise on AMPK signaling in skeletal muscle of subjects with type 2 diabetes: a time-course and dose-response study. Diabetes.

[31] Ko HJ, Zhang Z, Jung DY, Jun JY, Ma Z, Jones KE, Chan SY, Kim JK (2009). Nutrient stress activates inflammation and reduces glucose metabolism by suppressing AMP-activated protein kinase in the heart. Diabetes.

[32] Xie Z, Lau K, Eby B, Lozano P, He C, Pennington B, Li H, Rathi S, Dong Y, Tian R, Kem D, Zou MH (2011). Improvement of cardiac functions by chronic metformin treatment is associated with enhanced cardiac autophagy in diabetic OVE26 mice. Diabetes.

[33] Patel TP, Rawal K, Bagchi AK, Akolkar G, Bernardes N, Dias Dda S, Gupta S, Singal PK (2016). Insulin resistance: an additional risk factor in the pathogenesis of cardiovascular disease in type 2 diabetes. Heart Fail Rev.

[34] Pei H, Qu Y, Lu X, Yu Q, Lian K, Liu P, Yan W, Liu J, Ma Y, Liu Y, Li C, Li W, Lau WB, Zhang H, Tao L (2013). Cardiac-derived adiponectin induced by long-term insulin treatment ameliorates myocardial ischemia/reperfusion injury in type 1 diabetic mice via AMPK signaling. Basic Res Cardiol.

[35] Duan J, Guan Y, Mu F, Guo C, Zhang E, Yin Y, Wei G, Zhu Y, Cui J, Cao J, Weng Y, Wang Y, Xi M, Wen A (2017). Protective effect of butin against ischemia/reperfusion-induced myocardial injury in diabetic mice: involvement of the AMPK/GSK-3beta/Nrf2 signaling pathway. Sci Rep.

[36] Eriksson L, Nystrom T (2014). Activation of AMP-activated protein kinase by metformin protects human coronary artery endothelial cells against diabetic lipoapoptosis. Cardiovasc Diabetol.

[37] Gomez L, Paillard M, Thibault H, Derumeaux G, Ovize M (2008). Inhibition of GSK3beta by postconditioning is required to prevent opening of the mitochondrial permeability transition pore during reperfusion. Circulation.

[38] Juhaszova M, Zorov DB, Yaniv Y, Nuss HB, Wang S, Sollott SJ (2009). Role of glycogen synthase kinase-3beta in cardioprotection. Circ Res.

[39] Murphy E, Steenbergen C (2005). Inhibition of GSK-3beta as a target for cardioprotection: the importance of timing, location, duration and degree of inhibition. Expert Opin Ther Targets.

[40] Yin X, Zheng Y, Zhai X, Zhao X, Cai L (2012). Diabetic inhibition of preconditioning- and postconditioning-mediated myocardial protection against ischemia/reperfusion injury. Exp Diabetes Res.

[41] Zhou C, Bai J, Jiang C, Ye L, Pan Y, Zhang H (2017). Geranylgeranylacetone attenuates myocardium ischemic/reperfusion injury through HSP70 and Akt/GSK-3beta/eNOS pathway. Am J Transl Res.

[42] Min J, Wei C (2017). Hydroxysafflor yellow A cardioprotection in ischemia-reperfusion (I/R) injury mainly via Akt/hexokinase II independent of ERK/GSK-3beta pathway. Biomed Pharmacother.

[43] Mayerhofer M, Valent P, Sperr WR, Griffin JD, Sillaber C (2002). BCR/ABL induces expression of vascular endothelial growth factor and its transcriptional activator, hypoxia inducible factor-1alpha, through a pathway involving phosphoinositide 3-kinase and the mammalian target of rapamycin. Blood.

[44] Mottet D, Dumont V, Deccache Y, Demazy C, Ninane N, Raes M, Michiels C (2003). Regulation of hypoxia-inducible factor-1alpha protein level during hypoxic conditions by the phosphatidylinositol 3-kinase/Akt/glycogen synthase kinase 3beta pathway in HepG2 cells. J Biol Chem.

[45] Yao YY, Yin H, Shen B, Smith RS, Liu Y, Gao L, Chao L, Chao J (2008). Tissue kallikrein promotes neovascularization and improves cardiac function by the Akt-glycogen synthase kinase-3beta pathway. Cardiovasc Res.

[46] Bishop T, Ratcliffe PJ (2015). HIF hydroxylase pathways in cardiovascular physiology and medicine. Circ Res.

[47] Semenza GL (2014). Hypoxia-inducible factor 1 and cardiovascular disease. Annu Rev Physiol.

[48] Townley-Tilson WH, Pi X, Xie L (2015). The Role of Oxygen Sensors, Hydroxylases, and HIF in Cardiac Function and Disease. Oxid Med Cell Longev.

[49] Liu S, Ai Q, Feng K, Li Y, Liu X (2016). The cardioprotective effect of dihydromyricetin prevents ischemia-reperfusion-induced apoptosis *in vivo* and *in vitro* via the PI3K/Akt and HIF-1alpha signaling pathways. Apoptosis.

[50] Du M, Huang K, Huang D, Yang L, Gao L, Wang X, Li X, Wang C, Zhang F, Wang Y, Cheng M, Tong Q, Qin G, Wang L (2015). Renalase is a novel target gene of hypoxia-inducible factor-1 in protection against cardiac ischaemia-reperfusion injury. Cardiovasc Res.

[51] Marfella R, Esposito K, Nappo F, Siniscalchi M, Sasso FC, Portoghese M, Di Marino MP, Baldi A, Cuzzocrea S, Di Filippo C, Barboso G, Baldi F, Rossi F, D’Amico M, Giugliano D (2004). Expression of angiogenic factors during acute coronary syndromes in human type 2 diabetes. Diabetes.

[52] Jesmin S, Zaedi S, Shimojo N, Iemitsu M, Masuzawa K, Yamaguchi N, Mowa CN, Maeda S, Hattori Y, Miyauchi T (2007). Endothelin antagonism normalizes VEGF signaling and cardiac function in STZ-induced diabetic rat hearts. Am J Physiol Endocrinol Metab.

[53] Chou E, Suzuma I, Way KJ, Opland D, Clermont AC, Naruse K, Suzuma K, Bowling NL, Vlahos CJ, Aiello LP, King GL (2002). Decreased cardiac expression of vascular endothelial growth factor and its receptors in insulin-resistant and diabetic States: a possible explanation for impaired collateral formation in cardiac tissue. Circulation.

[54] Penumathsa SV, Thirunavukkarasu M, Samuel SM, Zhan L, Maulik G, Bagchi M, Bagchi D, Maulik N (2009). Niacin bound chromium treatment induces myocardial Glut-4 translocation and caveolar interaction via Akt, AMPK and eNOS phosphorylation in streptozotocin induced diabetic rats after ischemia-reperfusion injury. Biochim Biophys Acta.

[55] Lee SY, Ku HC, Kuo YH, Chiu HL, Su MJ (2015). Pyrrolidinyl caffeamide against ischemia/reperfusion injury in cardiomyocytes through AMPK/AKT pathways. J Biomed Sci.

[56] Yu L, Li Q, Yu B, Yang Y, Jin Z, Duan W, Zhao G, Zhai M, Liu L, Yi D, Chen M, Yu S (2016). Berberine Attenuates Myocardial Ischemia/Reperfusion Injury by Reducing Oxidative Stress and Inflammation Response: Role of Silent Information Regulator 1. Oxid Med Cell Longev.

[57] Yu L, Fan C, Li Z, Zhang J, Xue X, Xu Y, Zhao G, Yang Y, Wang H (2017). Melatonin rescues cardiac thioredoxin system during ischemia-reperfusion injury in acute hyperglycemic state by restoring Notch1/Hes1/Akt signaling in a membrane receptor-dependent manner. J Pineal Res.

[58] Xie H, Xu Q, Jia J, Ao G, Sun Y, Hu L, Alkayed NJ, Wang C, Cheng J (2015). Hydrogen sulfide protects against myocardial ischemia and reperfusion injury by activating AMP-activated protein kinase to restore autophagic flux. Biochem Biophys Res Commun.

[59] Patterson AJ, Xiao D, Xiong F, Dixon B, Zhang L (2012). Hypoxia-derived oxidative stress mediates epigenetic repression of PKCepsilon gene in foetal rat hearts. Cardiovasc Res.

[60] Zhang M, Yu LM, Zhao H, Zhou XX, Yang Q, Song F, Yan L, Zhai ME, Li BY, Zhang B, Jin ZX, Duan WX, Wang SW (2017). 2,3,5,4’-Tetrahydroxystilbene-2-O-beta-D-glucoside protects murine hearts against ischemia/reperfusion injury by activating Notch1/Hes1 signaling and attenuating endoplasmic reticulum stress. Acta Pharmacol Sin.

[61] Zhao GL, Yu LM, Gao WL, Duan WX, Jiang B, Liu XD, Zhang B, Liu ZH, Zhai ME, Jin ZX, Yu SQ, Wang Y (2016). Berberine protects rat heart from ischemia/reperfusion injury via activating JAK2/STAT3 signaling and attenuating endoplasmic reticulum stress. Acta Pharmacol Sin.

