# Supplementary material for: Diallyl trisulfide exerts cardioprotection against myocardial ischemia-reperfusion injury in diabetic state, role of AMPK-mediated AKT/GSK-3β/HIF-1α activation
Source: Oncotarget. 2017 Aug 24;8(43):74791–805. doi: 10.18632/oncotarget.20422 (PMC5650379; doi:10.18632/oncotarget.20422)
Supplement: Supplementary file 1 [file oncotarget-08-74791-s001.pdf]

Diallyl trisulfide exerts cardioprotection against myocardial ischemia-reperfusion injury in diabetic state, role of AMPK-mediated AKT/GSK-3β/HIF-1α activation

SUPPLEMENTARY MATERIALS

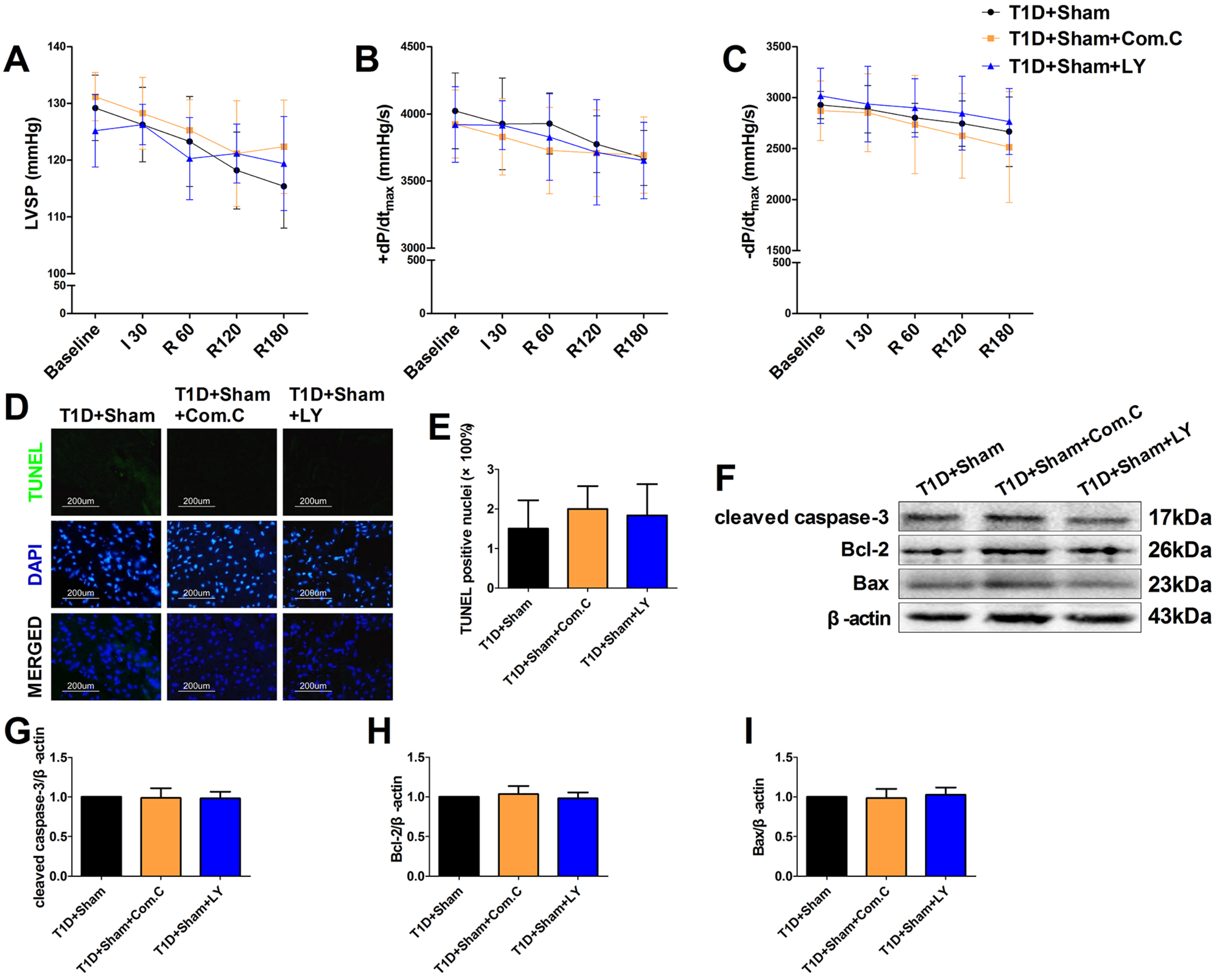

Supplementary Figure 1: Effects of Compound C and LY294002 on cardiac function and myocardial apoptosis in Sham-operated diabetic rats. **(A)**. Left ventricular systolic pressure (LVSP); **(B)** and **(C)**. The first derivative of left ventricular pressure (+dP/dt_{max} and -dP/dt_{max}). **(D)**. Representative photomicrographs of TUNEL staining (×200, bar=200μm). Apoptotic nuclei were stained with TUNEL (Row 1). Total nuclei were stained with DAPI (Row 2). **(E)**. Percentage of TUNEL positive nuclei; **(F)**. representative blots; **(G)**. cleaved caspase-3 expression; **(H)**. Bcl-2 expression; **(I)**. Bax expression. Data are expressed as mean ± SEM, n = 6 in each group. T1D, type 1 diabetes; Com.C, Compound C; LY, LY294002.

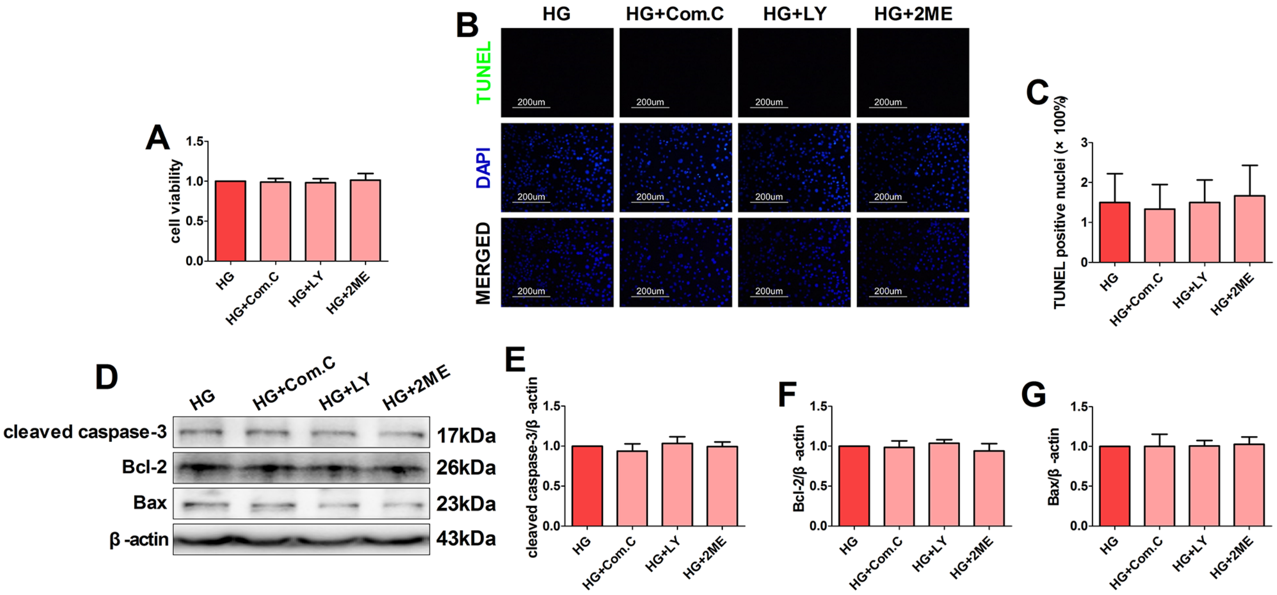

Supplementary Figure 2: Effects of Compound C, LY294002 and 2-methoxyestradiol on cellular viability and apoptosis in high glucose-treated H9c2 cardiomyoblasts. **(A)**. Cell viability. **(B)**. Representative photomicrographs of TUNEL staining (×200, bar=200μm). Apoptotic nuclei were stained with TUNEL (Row 1). Total nuclei were stained with DAPI (Row 2). **(C)**. Percentage of TUNEL positive nuclei; **(D)**. representative blots; **(E)**. cleaved caspase-3 expression; **(F)**. Bcl-2 expression; **(G)**. Bax expression. Data are expressed as mean ± SEM, n = 6 in each group. HG, high glucose; LY, LY294002; 2ME, 2-methoxyestradiol.
